# Ayurvedic herbal medicine and lead poisoning

**DOI:** 10.1186/1756-8722-4-51

**Published:** 2011-12-20

**Authors:** Krishna S Gunturu, Priyadharsini Nagarajan, Peter McPhedran, Thomas R Goodman, Michael E Hodsdon, Matthew P Strout

**Affiliations:** 1 Yale Cancer Center, Section of Hematology, Yale University School of Medicine, New Haven, CT, 06511 USA; 2 Department of Laboratory Medicine, Yale University School of Medicine, 55 Park Street, New Haven, CT, 06520 USA; 3 Department of Radiology, Yale University School of Medicine, 333 Cedar Street, New Haven, CT, 06520 USA

**Keywords:** Lead poisoning, basophilic stippling, anemia, Ayurveda

## Abstract

Although the majority of published cases of lead poisoning come from occupational exposures, some traditional remedies may also contain toxic amounts of lead. Ayurveda is a system of traditional medicine that is native to India and is used in many parts of world as an alternative to standard treatment regimens. Here, we report the case of a 58-year-old woman who presented with abdominal pain, anemia, liver function abnormalities, and an elevated blood lead level. The patient was found to have been taking the Ayurvedic medicine Jambrulin prior to presentation. Chemical analysis of the medication showed high levels of lead. Following treatment with an oral chelating agent, the patient's symptoms resolved and laboratory abnormalities normalized. This case highlights the need for increased awareness that some Ayurvedic medicines may contain potentially harmful levels of heavy metals and people who use them are at risk of developing associated toxicities.

## Background

Ayurvedic medicine is a traditional system native to India [1]. This system stresses the use of natural plant-based medicines, and minerals including sulfur, arsenic, lead, copper and gold are often added to formulations with the belief that these metals are essential components of vital molecules within the human body. In India, over 100 colleges offer degrees in traditional Ayurvedic medicine and in western countries, Ayurvedic medicine is gaining popularity as complementary treatment to modern medicine. Ayurvedic medicines are used to treat a wide spectrum of diseases from headaches to cancer. Currently, the United States does not specify a certification requirement for Ayurvedic practitioners, although many training programs are being offered through state-approved institutions. These practitioners are able to prescribe the medications and sometimes manufacture it themselves.

From 2000 to 2003, the Centers for Disease Control reported 12 cases of lead poisoning in adults associated with Ayurvedic medication intake occurring in five different states [2]. Some Ayurvedic preparations have been found to contain contained lead and/or mercury at 100 to 10,000 times greater than acceptable limits [3]. Although not common in western societies, lead exposure through dietary sources is a well-recognized phenomenon and in past years, calcium supplements have been a source of lead poisoning [4]. In addition to Ayurvedic medicine, other traditional medicines originating from Asian, Middle Eastern and Hispanic cultures have been found to contain lead and other heavy metals [5]. Although many health supplements are now subject to limited government regulation in the U.S. through the Dietary Supplement and Health Education Act of 1994, these medicines are readily obtainable as herbal remedies in health food stores and through the internet and their safety and efficacy are not regulated by government agencies such as the U.S. Food and Drug Administration (FDA) [6]. Thus, without sufficient public awareness, the risk of heavy metal exposure in individuals taking these supplements is quite high. Here, we present a case of lead poisoning secondary to ingestion of Indian Ayurvedic medicine, Jambrulin.

## Case presentation

A 58-year-old woman from India residing in the U.S. presented to the emergency department with a 10-day history of progressively worsening post-prandial lower abdominal pain and nausea accompanied by non-bilious and non-bloody vomiting. She was in her usual state of health prior to this illness. She had a past medical history of well-controlled non-insulin dependent diabetes mellitus and hypertension. A recent colonoscopy was unremarkable and she denied any history of melena or bright red blood per rectum. Physical exam was notable only for abdominal tenderness in the lower quadrants. Laboratory studies revealed a normochromic normocytic anemia with hemoglobin of 7.7 g/dL, hematocrit of 22.6%, MCV 87 fL and normal iron studies (Table 1). A CT scan of the abdomen and pelvis showed no specific abnormalities. The patient was discharged to home with anti-emetics and instructions to follow up with her primary care physician.

**Table 1:** Laboratory values

Lab values (Reference)	First emergency room visit	On admission	End of chelation
Hemoglobin (12-16 g/dL)	7.7	8.2	12.2

Hematocrit (37-47%)	22.6	23.5	36.4

Lead level (< 10 μ g/dL)	ND	102	46

Reticulocyte count (0.6-2.7)	ND	13.9	12.1

ZPP (15-36 μg/dL)	ND	912	ND

Total bilirubin (< 1.20 mg/dL)	1.34	1.35	0.66

AST (0-34 U/L)	34	54	30

ALT (0-34 U/L)	45	90	40

Abbreviations: ZPP, zinc protoporphyrin; ND, not done

**Figure 1:**
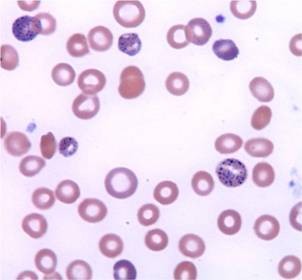
**Wright-Giemsa stained smear of peripheral blood**. Coarse basophilic stippling of the erythrocytes results from the poisoning of the 5' nucleotidase normally responsible for degrading RNA. Magnification is 1000x.

Five days later, the patient returned to the emergency department with worsening abdominal pain, nausea and bilious vomiting. Physical exam was remarkable for diffuse abdominal tenderness and pale conjunctivae. Laboratory evaluation was notable for anemia with hemoglobin of 8.8 g/dL, hematocrit of 23.5%, MCV of 87 fL and corrected reticulocyte count of 7%. The patient was admitted and subsequently underwent extensive evaluation for gastrointestinal abnormalities including esophagogastroduodenoscopy and colonoscopy, both of which were unremarkable. Review of the peripheral blood smear demonstrated normochromic, normocytic anemia with extensive coarse basophilic stippling of the erythrocytes (Figure 1). This triggered a screening for heavy metals, which revealed an elevated blood lead level (BLL) of 102 μ g/dL (normal < 10 μ g/dL). Zinc protoporphyrin (ZPP) was subsequently found to be elevated at 912 μ g/dL (normal < 35 μ g/dL). The clinical picture was consistent with lead poisoning. Upon further questioning, the patient disclosed that she had been taking an Indian Ayurvedic medicine called Jambrulin. The patient had obtained the medication from Unjha pharmacy through a family member in India. She had been taking 2 pills daily over a period of 5 to 6 weeks in an effort to enhance control of her diabetes. She stopped taking the medication approximately 2 weeks prior to admission because of the abdominal pain.

**Figure 2:**
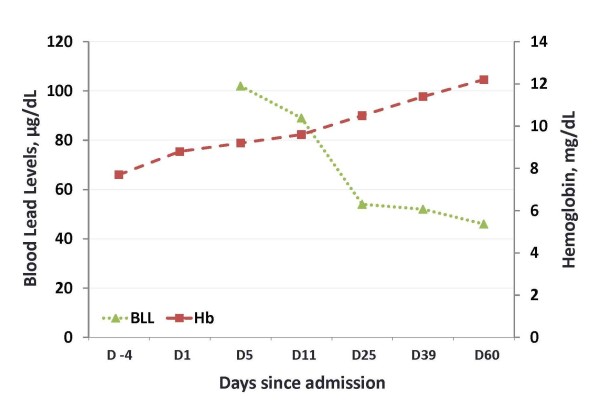
**Trends of hemoglobin and BLLs of the patient at diagnosis and during chelation therapy**. First emergency room visit corresponds to day -4; the day of admission during the second emergency room visit is designated as day 1.

The patient was instructed not to take Jambrulin and was started on dimercaptosuccinic acid (DMSA), an oral lead chelator at a dose of 10 mg/kg three times a day for five days followed by 10 mg/kg twice a day for two weeks. At the end of chelation therapy, her BLL decreased to 46 μg/dL, with improvement of her anemia and resolution of her abdominal pain (Figure 2 and Table 1). Of note, the patient's sister and brother had also been taking the same medication prior to our patient's diagnosis. They were subsequently found to have BLLs of 100 and 51 μg/dL, respectively and were advised to discontinue the supplement.

**Figure 3:**
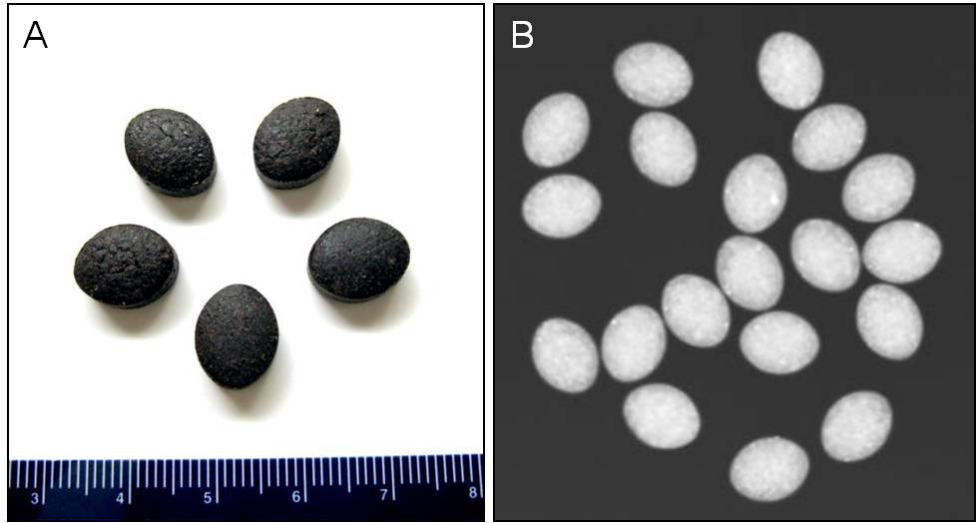
**Jambrulin tablets**. A. The tablets are 1.25 cm black, ovoid pills (ruler is in cm). B. X-ray shows diffusely opaque Jambrulin pills with flakes of high attenuating material.

Examination of the remaining medication from the patient revealed 1.25 × 1.0 cm ovoid black pills (Figure 3A). In addition, a radiographic study of the Jambrulin pills showed them to be diffusely opaque, containing flakes of high attenuating material, consistent with the presence of lead or other heavy metals (Figure 3B). One of the Jambrulin pills was crushed and solubilized in nitric acid and lead content was measured after serial 10-fold dilutions using a LeadCare II Blood Lead Test System as well as graphite furnace atomic absorption spectrophotometry. The pill tested was found to contain approximately 21.5 mg of lead. The pills were also sent to the Connecticut Department of Public Health Adult Blood Lead Epidemiology and Surveillance Program and Public Health Laboratory and were found to contain approximately 3.5% lead by weight or 35,000 μg/g.

## Discussion and Conclusion

Exposure to lead through ingestion or inhalation can occur from contaminated air, water, soil, food, and consumer products. One of the largest threats to children is lead-based paint that exists in many homes, especially those built in the U.S. before 1978. Occupational exposure is currently the most common cause of lead poisoning in adults. Findings of National Health and Nutrition Examination Surveys indicate that BLLs are in a continuous decline in all age groups and racial/ethnic populations [7]. Nonetheless, toxic exposure to lead through alternative sources remains a significant and poorly recognized public health problem.

Lead is stored in the blood, bone and soft tissues including the brain, spleen, kidneys, liver, and lungs. Like many other heavy metals, presence of excess levels of lead leads to production of free radicals which subsequently causes oxidative damage of cellular components including DNA and cell membranes [8]. Lead interferes with DNA transcription, enzymatic synthesis of vitamin D, and enzymes that maintain the integrity of the cell membranes. As an electropositive metal, lead has high affinity for negatively charged sulfhydryl groups resulting in denaturation of enzymes such as delta-aminolevulinic acid dehydratase (ALA-D) and ferrochelatase, both of which are important for heme synthesis. The disruption of heme synthesis leads to the accumulation of free erythrocyte protoporphyrins. Anemia often develops at very high BLLs (usually > 80 μg/dL). Inhibition of pyrimidine 5'-nucleotidase can prevent the degradation of ribosomal RNA in red blood cells leading to basophilic stippling on a peripheral smear, a classic finding which can be apparent at BLLs of ~50 μg/dL [9].

Currently, there are over 600 different Ayurvedic preparations that are manufactured for children and adults as herbal remedies to treat a wide range of illnesses including the common cold, diabetes, infertility, cardiovascular problems, psychiatric disorders, respiratory problems, rashes, and pain [3,10,11]. A comprehensive analysis of 193 Ayurvedic medications revealed the presence of heavy metals in ~20% of products analyzed [3]. Although lead was the most commonly detected heavy metal, many products also contained significant amounts of mercury and arsenic. Many of these medications are manufactured both in India and in the U.S. As they are marketed as supplements, they are not regulated by the U.S. FDA and are readily available in health food stores as well as over the internet.

Symptoms of adult lead poisoning are variable and include abdominal pain, nausea, constipation, anorexia, fatigue, decreased libido, headache, irritability, arthralgias, myalgias, anxiety and neurologic dysfunction ranging from subtle cognitive deficits to a predominantly motor peripheral neuropathy to encephalopathy. Some of these symptoms, especially the neurological symptoms, may be irreversible. The symptoms of lead toxicity usually appear at a BLLs of 40-60 μg/dL in adults [12]. Over the last 25 years, numerous cases of lead toxicity associated with Ayurvedic medicine have been reported in the literature (Table 2). Although the common denominator was the intake of Ayurvedic preparations, patients were taking these medications for a wide range of indications. The vast majority of individuals presented with gastrointestinal symptoms including abdominal pain, nausea, vomiting, anorexia and constipation and of them were found to have elevated BLLs. Managements varied widely and included oral chelation with D-penicillamine or DMSA or intravenous infusions of Ca-EDTA, Na-EDTA, or dimercaprol. In some instances, combination therapy was administered. The current reference range for acceptable BLLs in healthy individuals without excessive exposure to environmental sources of lead is < 10 μ g/dL for children and < 25 μ g/dL for adults [3]. To date, there are no clinical trials that define the optimal management although it is generally accepted that the first step is to identify and remove the source of the exposure. Chelation therapy should be initiated when the BLL is > 80 μ g/dL in asymptomatic and > 50 μ g/dL in symptomatic adults and should be continued until the BLL is < 50 μ g/dL.

**Table 2:** List of reported cases of lead toxicity associated with Ayurvedic mediation^1^

Reference	Ayurvedic Medicine	Age	Sex	Indication	Symptoms	Hgb (g/dL)	BLL (μg/dL)	Management
[2]	NR^2^	31	F	Fertility	Abdominal pain, nausea, spontaneous abortion	NR	112	Oral chelation^3^

[2]	Jambrulin	50	M	Diabetes	NR	NR	92	Oral chelation^3^

[2]	Jambrulin	40	F	Diabetes	NR	NR	92	Oral chelation^3^

[13]	NR	28	M	Weakness	Intestinal obstruction	8	145	Oral D-penicillamine

[14]	NR	23	M	NR	Anorexia, abdominal pain, weight loss	9.4	56	Ca -EDTA infusion

[15]	Vatyog Sahacharadi Gandharvahastadi	28	M	Back pain	Abdominal pain, constipation	NR	70	Oral DMSA

[16]	NR	58	M	Weakness	Paresthesias	14.3	74	Oral D-penicillamine

[17]	Guglu	41	M	Hypertension	Memory loss, anorexia, anhedonia	NR	161	Dimercaprol infusion

[18]	NR	51	F	Dengue fever	Memory loss, nausea, abdominal pain	NR	69	None

[19]	NR	24	M	Erectile dysfunction	Autonomic dysfunction, pseudo obstruction	NR	99	Oral DMSA

[20]	Multiple Ayurvedic medications^4^	39	F	Muscular dystrophy	Weakness, anorexia, constipation, back pain	7.9	88	Na-EDTA infusion and oral DMSA

[21]	Multiple Ayurvedic medicines^4^	35	F	NR	Abdominal pain, constipation, vomiting	8.3	140	Ca-EDTA infusion and oral DMSA

[22]	Multiple Ayurvedic medicines^4^	45	M	Anxiety	Weakness, vomiting and abdominal pain	14.2	122	Oral D-penicillamine

[22]	Gulkand	36	F	Psoriasis	Insomnia, headache, abdominal pain, joint pain	8	115	Ca-EDTA infusion

[22]	Chandraprabhavati EVR	46	M	Hand weakness	Abdominal pain	NR	42	None

^1^Cases that did not report BLL were excluded. ^2^Not reported. ^2^Oral chelation therapy was reported but specifics were not provided. ^4^Multiple medications were given but names were not provided.

In the case presented here, timely diagnosis and identification of source of exposure were critical in preventing the long-term consequences of lead poisoning. Both the FDA and the Connecticut Department of Public Health were notified and several additional cases of lead poisoning have since been attributed to this diabetic supplement. Despite the body of literature on this topic, lead poisoning through herbal supplements still remains a public health problem. Relatively few health care practitioners are familiar with the traditional medicines and health practices and most patients are unaware of the contents of herbal medications as well as the potential consequences of consuming these agents. Since patients often do not discuss the use of traditional medicines or herbal supplements, it is the responsibility of physician to obtain a detailed history of medications. Additional guidelines regulating the quality of dietary supplements are needed. Enhancing public awareness about the harmful effects of the seemingly innocuous herbal supplements is essential for the prevention of heavy metal poisoning.

## Consent

Written informed consent was obtained from the patient for publication of this Case report and accompanying images. A copy of the written consent is available for review by the Editor-in-Chief of this journal.

## Competing interests

The authors declare that they have no competing interests.

## Authors' contributions

KSG and PM managed the patient as an outpatient and drafted the manuscript. PN and MEH performed the laboratory analysis of the Jambrulin pills and edited the manuscript. TRG performed the radiographic analysis of the Jambrulin pills. MPS managed the patient in the hospital and contributed to critical revisions of the manuscript. All authors read and approved the final manuscript.
